# Researchers’ First Duty Is to the Participants

**DOI:** 10.1200/JGO.2016.005058

**Published:** 2016-05-18

**Authors:** Sandhya Srinivasan, Bebe Loff, Amar Jesani, Veena Johari, Sarojini N

**Affiliations:** **Sandhya Srinivasan**, Indian Journal of Medical Ethics, Mumbai, India; **Bebe Loff**, Michael Kirby Centre for Public Health and Human Rights, Melbourne, Australia; **Amar Jesani**, Indian Journal of Medical Ethics, Mumbai, India; **Veena Johari**, Indian Journal of Medical Ethics, Mumbai, India; **Sarojini N**, Jan Swasthya Abhiyan, New Delhi, India.

## To the Editor:

In their recent article in *Journal of Global Oncology*, on the ethics of clinical trials in low-income countries, Prasad et al^1^ state that where a proven intervention exists, placebo-controlled trials may nonetheless be ethical if the intervention under study has a reasonable chance of being implemented in the host community. To illustrate their point, they discuss a cluster randomized controlled trial^2^ in Mumbai, India, of visual inspection with acetic acid (VIA) by trained health workers to screen for cervical cancer. The efficacy of VIA as a screening method had already been confirmed in many studies.^3^

The Mumbai trial, largely funded by the National Institutes of Health, commenced on September 30, 1997, and followed women until the end of 2015. The trial had a no-screening arm, and the end point was mortality from cervical cancer.

The year 1997 was also when Lurie and Wolfe published their now famous paper^4^ titled “Unethical Trials of Interventions to Reduce Perinatal Transmission of the Human Immunodeficiency Virus in Developing Countries,” in which they challenged the use of placebo-controlled trials in developing countries when a treatment is available in the country funding the research. In that paper, they note that “Some officials and researchers have defended the use of placebo-controlled studies in developing countries by arguing that the subjects are treated at least according to the standard of care in these countries, which consists of unproven regimens or no treatment at all. This assertion reveals a fundamental misunderstanding of the concept of the standard of care.”^4(p854-855^^)^

The absence of care, they point out, is not a standard of care. The standard is the effective treatment that should be provided, regardless of whether it is universally available in the host country.

In the current case, although the researchers knew that some women in the no-screening arm would die precisely because they were not screened, the authors argue that the placebo arm was permissible because no universal cervical cancer screening program exists; thus, “no treatment” may be regarded as the local standard of care.

The standard screening method, the Papanicolau test, has been available and offered to women free of charge in tertiary public hospitals in Mumbai and in other cities since the early 1970s, including in the Tata Memorial Hospital, which conducted the VIA trial with a no-screening arm.

We suggest that there are critical ethical concerns that the study authors would have become aware of from the year 1997 onward. The Declaration of Helsinki (DoH) in 2000^5^ took a firm stand on this matter, and subsequently in 2001^6^ provided a clarification that instead resulted in confusion, remedied in 2008. Article 33 of the current DoH on the use of placebo reflects the position adopted by the DoH since 2008. It states: “...patients who receive any intervention less effective than the best proven one, placebo, or no intervention will not be subject to additional risks of serious or irreversible harm as a result of not receiving the best proven intervention.”^7^

Furthermore, longstanding ethical principles are neatly summarized in the DoH of 2013.^7^ Articles 8 and 9 state that, “while the primary purpose of medical research is to generate new knowledge, this goal can never take precedence over the rights and interests of individual research subjects”; and that “it is the duty of physicians who are involved in medical research to protect the life … of research subjects. The responsibility for the protection of research subjects must always rest with the physician or other health care professionals and never with the research subjects, even though they have given consent.”

The substance of the issues addressed by these Articles has not changed greatly over time.

Taken together, it would seem that when death is a foreseeable outcome for participants and it is possible to prevent death, the potential for social good should not be the overwhelming consideration. This principle lies at the heart of research ethics. When compared with this, the justifications given for the study design in this case and in other similar trials of VIA^8,9^ fall disturbingly short.

We agree with the authors that there is a need to design and conduct research that is locally relevant and will be used to benefit the community. However, the importance of the research does not permit putting the participants at foreseeable risk of significant harm.
